# The role of care-seeking behavior and patient communication pattern in online health information-seeking behavior - a cross-sectional survey

**DOI:** 10.11604/pamj.2022.42.124.33623

**Published:** 2022-06-15

**Authors:** Martin Gameli Akakpo

**Affiliations:** 1 Department of Human Development and Psychology, Faculty of Arts and Sciences, Regent University College of Science and Technology, Accra, Ghana

**Keywords:** Information-seeking behaviour, eHealth, care-seeking behaviour, health care surveys, statistical models, patients, surveys

## Abstract

**Introduction:**

online health information-seeking behaviour has been on the increase and patients are seeking more responsibility in decisions about their health. Previous studies have mostly predicted online health information-seeking behaviour with demographic characteristics and not current behaviours driven by improving online information. The study attempts to bridge the gap between fast-developing patient online health information-seeking behaviour and health behaviour research.

**Methods:**

a cross-sectional survey was conducted in the Ghanaian city of Accra. Descriptive statistical analysis was used to reveal frequencies and range of scores on measurement scales. All measurement scales used in the study were tested for reliability. Univariate analysis was used to find correlations and key properties of the dependent and independent variables. Multivariate analysis was used to test hypotheses.

**Results:**

one hundred and eighteen (118) adults were surveyed. Cronbach alpha reliabilities of measurement scales ranged from .71 to .91. The hypothesis that care seeking behaviour and patient communication pattern predict online health information seeking behaviour, was supported (r = .45, standard error = 5.84) by a multiple linear regression.

**Conclusion:**

patients are engaged in online health information seeking because they want to be more active in decisions about their health. Findings further suggest that patients rely on online information to direct communications with their caregivers.

## Introduction

The study of eHealth literacy, care-seeking behaviour, preferred online sources, patient communication patterns, and online health information-seeking behaviour (OHISB) in a single study introduces a helpful perspective in studies of patient intentions. Ongoing shifts in online information availability are influencing patient behaviour [1]. The traditional approach where patients trust their caregivers and caregivers ‘enjoy’ complete control during interactions with patients is fast changing. A growing body of research suggests that patients actively search for information as soon as they notice symptoms and now have a higher sense of responsibility. Even when they visit their caregivers, some patients compare medical advice to online sources and seek advice from other internet users. Research about online health information-seeking behaviour (OHISB) has been gathering pace globally with some recent attempts in health systems such as India [2], Italy [3], United States [4], China [5] and Ghana [6].

These days ‘Dr. Google’ has become a prominent source for patients to quickly check symptoms and find the best specialist around them [7]. Despite the concerns healthcare professionals may have against online health information, its use by patients continues to increase [8]. It has become inevitable to some healthcare professionals that credible information needs to be made available online and patients promptly directed to seek care from experts [9]. This however requires studies to update caregivers about OHISB and how it can change events in their consulting rooms and wards. Van Riel *et al*. [7], suggest that online health information is not a threat to the authority of the caregiver but a tool to create mutual understanding throughout the care process. Despite the laudable attempts to improve knowledge about OHISB across many health systems, it will still be beneficial for studies like the current one to seek additional perspectives and use different samples. The next section defines the problem identified by the researcher.

**The problem:** there is a discrepancy between the pace at which patient behaviours are changing and the direction of research on OHISB [7]. Even more urgent is the awareness and readiness of caregivers to accept this new patient dimension. This study is designed to add a new perspective to the prediction and understanding of OHISB. Previous studies mostly focused on predicting OHISB with demographics other than their actual visits to health professionals and the resulting conversations with their caregivers [9].

The problem with the approach captured in Li *et al*. [9], is that demographic characteristics do not adequately account for the intention and active role patients now seek. Patient care-seeking behaviour and communication patterns are more within their immediate control unlike demographic variables like gender, educational status and age. In addition to addressing the problem of demography-biased predictions, this study captured the current information-seeking behaviours of the digital age [1] and related them to patients´ care-seeking behaviour and communication patterns. The approach contributes by reducing the gap between patients OHISB and the understanding of both researchers and health practitioners. To clarify the scope of this study, the next paragraphs briefly describe each of the investigated variables with a focus on their definition, measurement, and current use in literature.

**eHealth literacy:** covers the ability of individuals to find and use online health information [10]. An eHealth literacy measurement scale developed by Norman and Skinner [11], focuses on knowledge, confidence and skills patients need to seek, find and use online health information. Recent studies such as [1] and [12] have supported the importance of eHealth literacy in safe health practices, health knowledge, and treatment compliance.

**Online Health Information Seeking Behaviour (OHISB):** is the intentional search of information to fill a knowledge gap about a specific health topic [2]. OHISB contains cognitive and emotional components [13]. This behavior includes searches about many health topics such as dieting, exercise, and treatment [13]. Adjei Boakye *et al*. [6] separated these into information seeking on health or wellness on one hand and information about managing chronic conditions such as cancer on the other hand.

**Care-seeking behaviour:** involves finding solutions to a health challenge [14]. Knowledge of care-seeking options and actual care-seeking have been recently investigated with support for improving information about care options [15]. The concept is framed in several ways including health-seeking behaviour [16], medical advice-seeking behaviour [17], help-seeking behaviour [15] and treatment-seeking behaviour [18]. To scope, this work considered all acts that seek to solve a health problem by visiting a hospital [19], as care-seeking behaviour. The visibility of these treatment options and centres has been significantly enhanced by the internet due to the availability of digital maps, catalogues, and other online resources [1, 20].

**Preferred sources of online information:** eHealth literacy, OHISB and the use of the internet to find the best care influenced by the preference patients have for a specific source amongst the diverse channels [5, 20]. Preference in the context of this study means the belief that information obtained online is credible, easy to access and useful [20, 21]. The credibility of online sources is of interest to both patients and medical professionals [7]. In recent years, medical professionals have attempted to improve their online visibility through institutional websites, blogs, podcasts, and other channels. An aim has been to improve the availability of credible information for patients in search of useful information.

**Patient communication pattern (PCP):** is concerned with the nature of the relationship between a patient and their healthcare professional [7]. This includes the degree to which the health professional delivers clear information and the extent to which the patient is involved in the conversation [22]. A measure of PCP by Ilan and Carmel [22], considers dimensions of communication including the patients´ quality of life, fears and other related emotions. Research has suggested that the relationship between patients and doctors has moved from an authoritative or instructional style to a collaboration [22]. Tan and Goonawardene [8] use the term ‘internet-informed patients’. Van Riel *et al*. [7], acknowledged the influence ‘Dr. Google’ now exerts on patient perceptions, care-seeking behaviour, and communication during visits to their caregivers.

**The objectives:** the gap between patient behaviour and research supports the need for this study. Despite Global attempts to improve literature about OHISB, the pace of patients´ behavioural change has been faster. The main objective of the study is to predict OHISB. The second objective is to rank Online sources of health information. The study also tests the relationship between eHealth literacy and OHISB. This relationship has been suggested by some previous studies and with a new sample, this study seeks to test those findings in another health system. The following summarizes the study objectives: explain the relationship between eHealth literacy and OHISB; explain the roles of care-seeking behaviour and patient communication pattern in patients´ OHISB; rank sources preferred by patients for online health information; present findings using a different sample and enrich literature about OHISB; test scales for OHISB, eHealth literacy, patient communication pattern and care-seeking behaviour in a different setting and sample. The study made two predictive statements about the dependent variable (OHISB).

**Hypotheses:** e-Health Literacy is related to OHISB. OHISB is predicted by care-seeking behaviour and Patient communication pattern. A research question explored the preferences of patients for selected online sources of health information. These sources were selected because of suggestions in literature about their use by patients for information-seeking. This research question makes no directional statements because of the exploratory objective for which it was developed.

**Research question:** which online sources are most preferred by patients?

## Methods

**Study design:** a cross-sectional survey was conducted to test some determinants of online health information seeking behaviour in some Ghanaian adults. The competence of patients in online information search and their preferred sources of online health information was additionally tested.

**Setting:** the study was conducted in Accra, the capital city of Ghana in West Africa. Accra is the most populated and modern city in Ghana, with an estimated population of 5.4 million according to Ghana´s Statistical Service. Most of these inhabitants are however informal workers and people with little access to literacy in online health information libraries. Some younger and more literate adults use online sources to investigate symptoms, find hospitals or inform themselves about possible treatments before they visit their caregivers. The study was conducted between February and June 2021 using both online and paper-pen versions of a questionnaire. Paper-pen versions were distributed in class or a link to the questionnaire was sent to them on WhatsApp messenger. Respondents needed 8-10 minutes to complete each questionnaire. This was done at the end of lectures and students were not allowed to take questionnaires out of the lecture hall, to ensure that responses were provided under the same condition.

**Participants:** the study population was adults who are resident in Accra, who owned a smartphone and had regular access to the internet. The selection of participants was done in lecture halls after a brief introduction to the purpose of the study. All participants who agreed to complete the questionnaires were given a copy (paper-pen) and those who opted to respond online were sent a link to the online survey.

**Variables:** the outcome variable in the study was online health information seeking behaviour (OHISB). The predictor variables were care seeking behaviour and patient communication pattern. eHealth literacy and preferred sources of online health information were additional variables of interest. eHealth literacy measured the self-efficacy of the patient regarding online information search and the quality of their judgements about the accuracy of such information. OHISB, measured the intention of participants to search for health information online to fill their knowledge gap. Care seeking behaviour in this study measured the reasons which prompt participants to visit a health centre. Patient communication pattern measured the nature of communication between participants and their caregivers. Preferred sources of online health information measured the level of confidence participants had in selected online sources of health information.

### Data sources/measurement

***Data collection:*** online data was collected through an online survey deployed on Nubia Metrics. Paper-pen versions of the questionnaire were manually inputted into SPSS and analysed together with the data from the online version of the survey.

***Data collection tool:*** the questionnaire comprised of age and place of residence as demographic questions and five scales, namely the e-health literacy scale, online health information-seeking behaviour (OHISB) scale, a care-seeking behaviour measure, a preferred online sources list and a patient communication pattern scale. eHealth literacy was measured with a scale developed by Norman and Skinner [11]. In this study, the 8-item scale (Annex 1) was presented on a 5-point Likert ranging from ‘Strongly Disagree’ to ‘Strongly Agree’. OHISB was measured with an 8-item instrument which was presented on a 5-point Likert scale (Annex 1) and was adopted from Wong and Cheung [1]. Care-seeking behaviour was measured with a 5-item scale (Annex 1) which was derived from previous studies on care-seeking behaviour [17, 19] and presented a 5-point scale ranging from ‘Never’ to ‘Always’. Patient communication pattern was measured with a scale developed by Ilan and Carmel [22]. This was presented on a 5-point Likert scale ranging from Strongly Disagree to Strongly Agree. To measure preferred sources of health information, a 5-item likert scale (Annex 1) with scores ranging from ‘Poor’ to ‘Excellent’ was used.

**Bias:** to address potential sources of bias, the researcher did not include participants who were medical professionals and medical students or people who had daily exposure to the hospital setting because of their work.

**Study size:** the sample size was calculated based on the estimated population of students at the two selected universities, who were in undergraduate programs and were regular users of the internet. Using an estimated population of 3000 with a 95% confidence interval and a 5% margin of error. This smaller population was used because of the demographic similarities (age and digital literacy) of university students and the limitation of time/resources. 118 responses were obtained from 150 invitations, this resulted in a response rate of 78.67%.

**Quantitative variables and statistical methods:** all variables used in analysis were quantitative and were measured with standard scales. These scales were tested for reliability with Cronbach alpha reliability (Table 1). To control the quality of data, results and interpretations, returned questionnaires with missing data were excluded from the final data entry. All data was entered into IBM SPSS version 26. Univariate analysis was used to test each variable for the frequency, mean, standard deviation, reliability and range of scores (Table 1). Multivariate analyses commenced with a Pearson product moment correlation coefficient, which was used to test the strength of the correlation between all the variables. To test the assumption of multicollinearity, the Pearson test was used to measure the existence of only a weak or no correlation between the variables to be included in the multivariate model. A multiple linear regression was used to test the hypothesis that OHISB can be predicted by patient communication patterns and care seeking behaviour (Table 2, Table 3). The research question investigating the preferred sources of online health information was answered with a ranking of most preferred to least preferred sources (Table 4). The measure of preferred sources of online health information was included for exploratory reasons and was thus not tested in the multivariate model. eHealth literacy was included to measure the levels of digital health literacy of all participants, it was not included in multivariate analysis. The multivariate model included OHISB as an outcome variable, with patient communication pattern and care seeking behaviour as predictors.

**Table 1:** descriptive statistics, correlations, and reliabilities of scores obtained on eHealth literacy, OHISB, trust in online sources, care-seeking behavior, and patient communication pattern

Variables	L/H	M	SD	1.	2.	3.	4.	5.
1. e-Health literacy	8/40	26.77	6.43	(.90)				
2. Online health info seeking	8/40	24.01	6.45	.45*	(.87)			
3. Trust in online sources	7/30	20.05	5.04	.34*	.27*	(.84)		
4. Care seeking behavior	5/22	14.38	3.58	.30*	.41*	.29*	(.71)	
5. Patient communication patterns	13/60	37.96	9.72	.33*	.32*	.25*	.36*	(.91)

Note: L/H= Lowest score/Highest score, M= Mean. SD = Standard deviation, * = significant (p) < 0.05, ( ) = Cronbach´s alpha (α) reliability

**Table 2:** ANOVA table of OHISB predicted by care-seeking behavior and patient communication pattern

	Sum of Squares	df	Mean Squares	F	Sig (p)
Regression	983.48	2	491.74	14.40	.00
Residual	3825.45	112	34.16		
Total	4808.92	114			

Note: df = degrees of freedom, F = F-ratio, Sig (p) = Significance (p-value). The table shows summaries of an ANOVA computed to predict OHISB.

**Table 3:** coefficients table of the predictors of OHISB

Variables	B	SE	β	Sig (p)
Constant	10.07	2.67		
Care seeking behavior	.14	.06	.21	.02
Patient communication pattern	.61	.15	.34	.00

Note: B = Unstandardized coefficient, SE = Standard Error, β = Beta, Sig (p) = Significance (p-value).

**Table 4:** a rank of scores obtained on preferred sources of online health information

Sources	Sum	Mean	SD
Webpages of prominent medical institutions	383	3.30	1.11
Blogs by medical doctors	368	3.17	1.03
Health-linked NGOs	332	2.89	.94
Social media posts by medical professionals	324	2.79	1.03
Advertisements by private clinics	312	2.69	.85
Online patient discussion groups	304	2.62	.98
Government websites and blogs	303	2.61	1.11

Note: SD = Standard Deviation

**Ethical considerations:** the first page of the questionnaire explained the purpose of the study to all respondents. The principle of informed consent was adhered to, and the confidentiality of responses was assured. All respondents were aware that they could stop filling the questionnaire at any time if they had any concerns. The researcher informed academic superiors about the conduct of the study and sought advice on whether an approval was necessary at any stage. Due to the absence of a negative impacts, the short duration and the insensitive nature of the data and ethics approval was not required. No personal data of participants was collected. Online participants received links from their class leaders under the supervision of the researcher with the receipt of any personal data.

## Results

**Participants:** a total of 118 adults aged between 18 and 39 [mean age = 22.53, standard deviation = 3.23] participated in the study. After initial data entry, two cases were excluded from final analysis due to missing data. These 2 participants failed to complete all items on some questionnaires and were thus excluded from both univariate and multivariate analyses.

**Descriptive data:** Cronbach´s alpha reliabilities computed for all the scales used in the study showed acceptable consistencies from .71 to .91, as presented in Table 1. Scores on Online health information seeking behaviour (n = 117) ranged from 8 to 40 with a mean of 26.77 and a standard deviation of 6.43. On eHealth literacy (n = 116), scores ranged from 8 to 40 with a mean of 24.01 and a standard deviation of 6.47. Care seeking behaviour (n = 118) showed a mean score of 14.38, a standard deviation of 3.58 with scores ranging from 5 to 22. Scores on patient communication pattern (n = 118) ranged from 13 to 60 with a mean of 37.96 and a standard deviation of 9.72.

### Main results

**eHealth Literacy is related to OHISB:** a Pearson product-moment correlation coefficient supported this hypothesis, with a significant positive correlation (Table 1).

**OHISB is predicted by care-seeking behaviour and Patient communication pattern:** a multiple linear regression was used, and a significant model emerged in support of the hypothesis. The model summary results were *r= .45, r-square= .21, Adjusted r-square = .19 with a standard error of 5.84*.As part of the regression analysis, an ANOVA (Table 2) and a coefficients table (Table 3) indicated that the variances were caused by scores on care-seeking behaviour and patient communication pattern. Both hypotheses tested in the study were accepted.

**Research question:** *Which online sources are most preferred by health information seekers?* Rankings of both sum scores and means placed webpages of prominent medical institutions highest, blogs by medical doctors second and health-linked NGOs third. Social media posts, advertisements by private clinics, and online patient discussion groups placed 4^th^, 5^th^, and 6^th^, respectively (Table 4). The least preferred source was government websites and blogs. The meaning of these results concerning patient behaviour, medical practice, and research are discussed in the next chapter.

## Discussion

**Key results:** firstly, the study findings suggest that health-information literacy is related to OHISB. A positive relationship in this context means competence in online information search will correspond with competence in bridging a patient´s knowledge gap about their health. This is like findings by Wong and Cheung [1]. In other words, if patients feel confident that they can find health information online, they are more likely to find information about their symptoms, where to seek care, and other relevant health information.

Secondly, patient communication patterns and care-seeking behaviour predict OHISB. This means that the patients´ intention to seek care from professionals and be involved in the decisions made by caregivers influences their intention to find health information online. Engaging in OHISB before healthcare visits increases the likelihood that patients will have a better understanding of their condition and ask important questions when they meet the health professional. This is like findings by Tan and Goonawardene [8]. From these findings, patients intentionally engage in OHISB to solve urgent healthcare problems and assume more control. It is therefore advisable for healthcare professionals to encourage patients. Caregivers should support more efficient patient behaviour by suggesting accurate sources of information and providing guidance about how to use it.

Finally, the study found web pages of medical institutions to be the most preferred source of online information. In recent years, prominent institutions such as Mayo Clinic, Cleveland and many others have established a visible online presence. These websites describe symptoms of some conditions and suggest how health professionals may treat them. Efforts by these institutions and all healthcare players are laudable, but more effort can be directed at helping patients to decide when and where to seek care. Additionally, caregivers can suggest credible online sources to patients if they are interested in knowing more about their condition or the mutually agreed treatment.

The findings from this study have provided an additional understanding of OHISB. To summarize, there is a suggestion that OHISB is now an important driver of responsible patient behaviours, that is, seeking care, being involved in the diagnosis and treatment planning. A basic feature of efficient OHISB is eHealth literacy. This makes both eHealth literacy and OHISB important patient characteristics that should be improved through caregiver suggestions and programs to aid responsible patient behaviour.

**Limitations:** firstly, this study was conducted with small sample size and in one city, future studies can use a larger sample size across multiple cities to allow more generalizability. Secondly, future works can investigate the reasons for the rankings of online sources by the patients. Of particular interest are the low rank of official government websites and government blogs.

**Interpretation:** the findings have implications for health communication researchers and medical practitioners. For researchers, findings further extend the growing perspective that patients desire to be more active and responsible in decisions taken when they visit healthcare centres. This calls for more research to provide clarity on the exact active role patients want to play when they visit caregivers. For medical practitioners, this study advises that some patients who visit healthcare centres rely on online health information to ask questions about their diagnoses and treatment. These behaviours may sound intrusive and non-traditional but are increasing amongst adults who have access to the enormous information on the internet. Caregivers can view this OHISB as an increased sense of responsibility from patients.

**Generalizability:** findings from the study can be generalized to the population of adults who are regularly seek health information online. Interpretations can extend to caregivers who interact with such ‘google-informed’ patients and must deal with regular questions about symptoms and treatment options. Prominent medical institutions in Ghana and other countries can benefit from the findings by updating their websites and other online sources with up-to-date health information such as symptoms, treatment and care seeking advice.

## Conclusion

This study adds to calls for caregivers to recognize the active role patients want to play in their health. Their intentions to seek care and communicate with control influences their decisions to seek information online. To improve outcomes in healthcare, it will be useful for caregivers and researchers to encourage OHISB and help improve patient eHealth literacy. This like the views of Van Riel *et al*. [7] which suggests that OHISB is not a threat to good healthcare but an asset.

### What is known about this topic

- *Health information is now easy to access online;*
- *Before visiting caregivers, most tech-savvy patients read extensively about their symptoms online;*
- *Online health information seeking behaviour can be predicted by demographic factors such as age and educational status*.

### What this study adds

- *The online health information seeking behaviour (OHISB) of patients is predicted by their care seeking behaviour and how they communicate with caregivers;*
- *Caregivers are advised to embrace the active participation of patients in the care process and view it as an act of mutual understanding; Web pages of prominent medical institutions most preferred by patients as a source of credible health information;*
- *Studying patients´ communication pattern, care-seeking and online health information seeking in one study, introduces a comprehensive and new perspective*.

## Annex

**Annex 1**: questionnaire used for data collection, pdf (508 Ko)
